# Exceptional Strengthening of Biodegradable Mg-Zn-Ca Alloys through High Pressure Torsion and Subsequent Heat Treatment

**DOI:** 10.3390/ma12152460

**Published:** 2019-08-02

**Authors:** Jelena Horky, Abdul Ghaffar, Katharina Werbach, Bernhard Mingler, Stefan Pogatscher, Robin Schäublin, Daria Setman, Peter J. Uggowitzer, Jörg F. Löffler, Michael J. Zehetbauer

**Affiliations:** 1 Physics of Nanostructured Materials, Faculty of Physics, University of Vienna, 1090 Vienna, Austria; 2 Center for Health & Bioresources, Biomedical Systems, AIT Austrian Institute of Technology GmbH, 2700 Wiener Neustadt, Austria; 3 Department of Physics, GC University, 54000 Lahore, Pakistan; 4 Laboratory of Metal Physics and Technology, Department of Materials, ETH Zurich, 8093 Zurich, Switzerland; 5 Institute of Nonferrous Metallurgy, Montanuniversität Leoben, 8700 Leoben, Austria

**Keywords:** Mg alloy, Mg-Zn-Ca, severe plastic deformation (SPD), high pressure torsion (HPT), dislocation loops, precipitates, vacancies

## Abstract

In this study, two biodegradable Mg-Zn-Ca alloys with alloy content of less than 1 wt % were strengthened via high pressure torsion (HPT). A subsequent heat treatment at temperatures of around 0.45 *T*_m_ led to an additional, sometimes even larger increase in both hardness and tensile strength. A hardness of more than 110 HV and tensile strength of more than 300 MPa were achieved in Mg-0.2Zn-0.5Ca by this procedure. Microstructural analyses were conducted by scanning and transmission electron microscopy (SEM and TEM, respectively) and atom probe tomography (APT) to reveal the origin of this strength increase. They indicated a grain size in the sub-micron range, Ca-rich precipitates, and segregation of the alloying elements at the grain boundaries after HPT-processing. While the grain size and segregation remained mostly unchanged during the heat treatment, the size and density of the precipitates increased slightly. However, estimates with an Orowan-type equation showed that precipitation hardening cannot account for the strength increase observed. Instead, the high concentration of vacancies after HPT-processing is thought to lead to the formation of vacancy agglomerates and dislocation loops in the basal plane, where they represent particularly strong obstacles to dislocation movement, thus, accounting for the considerable strength increase observed. This idea is substantiated by theoretical considerations and quenching experiments, which also show an increase in hardness when the same heat treatment is applied.

## 1. Introduction

The first observations of the corrosion and degradation behavior of magnesium in the human body were made over a hundred years ago [1]. In recent times, interest in this topic increased because biodegradable implants made of metallic materials—in contrast to biodegradable polymers—may also be used in load-bearing applications [2,3,4,5,6,7]. According to Gunde et al. [8], the requirements for biodegradable materials in osteosynthesis applications are (i) high strength and reasonable ductility; (ii) slow and homogeneous degradation (inhomogeneous degradation may lower the load-bearing capacity of the implant and fast degradation would result in a high rate of hydrogen release, which can have detrimental effects on the surrounding tissue [9,10]); and (iii) material release, which is not harmful to the human body. The alloy composition is crucial for all these points.

Pure Mg exhibits not only very good biocompatibility but also a properly low degradation rate [11,12]. However, its strength is limited. One way to increase the latter is alloying, but alloying elements usually form precipitates that are nobler than the Mg matrix. These particles act as cathodic sites for micro-galvanic corrosion and thereby strongly increase the degradation rate [13,14]. A possible solution to this dilemma is to use only limited amounts of highly biocompatible alloying elements and to strengthen the alloy using plastic deformation, i.e., grain boundary and dislocation strengthening mechanisms. 

Applying methods of severe plastic deformation (SPD) [15,16,17] such as high pressure torsion (HPT) [18] or equal channel angular pressing (ECAP) [19] to crystalline solids leads to the formation of ultra-fine grained or even nanocrystalline materials with a high concentration of lattice defects and considerably increased strength. As Mg has a hexagonal closed-packed (hcp) lattice structure, the primary deformation mechanism is basal slip. Due to the big difference in the critical resolved shear stress between primary and secondary slip, plastic deformation of Mg is generally limited—especially at low temperatures [20]. Therefore, SPD-processing of Mg is usually performed at elevated temperatures. A further consequence of the hcp lattice is the texture dependence of strength and formability [21,22,23]. Due to the rather low melting temperature (*T*_m_ = 650 °C), dynamic recrystallization plays a significant role during SPD-processing of Mg [24,25]. It is also reported that the grain refinement mechanism is different from that in face-centered cubic (fcc) metals [25,26].

Most studies on SPD-processing of Mg alloys (and sometimes also pure Mg) thus far have been carried out via ECAP (see for example references [27,28,29,30,31,32,33,34]). ECAP of Mg alloys is usually performed at temperatures above 200 °C to avoid the formation of cracks [26]. Under these conditions, the grain refinement is rather limited due to strong dynamic recovery and recrystallization effects [22,35,36], and grain sizes below 1 µm are usually not achieved. Smaller grain sizes can only be realized using elaborate ECAP procedures where the temperature is reduced in steps for each pass [25,37]. 

In contrast to ECAP, HPT-processing of Mg alloys, and of pure Mg, can be performed at room temperature without the formation of cracks, and thus does not require pre-processing [38,39,40,41,42,43]. The result is considerable grain refinement, and grain sizes in HPT-processed Mg alloys can be as small as 100 nm [44,45].

It is the aim of this study to investigate the strengthening capability of biodegradable Mg alloys with low alloy content through grain refinement via severe plastic deformation using high pressure torsion. The Mg-Zn-Ca system chosen for this study is a very promising candidate for biodegradable implants because both alloying elements are mineral nutrients and therefore not harmful to the human body. With Mg, Ca forms precipitates that prohibit grain growth during solidification and metallurgical treatments at higher temperatures, and thereby increases the strength of the initial cast or extruded material [46]. Ca is, in addition, even less noble than Mg, which—according to Hofstetter et al. [47]—generates a desirably slow and homogeneous degradation behavior in low alloyed Mg-Zn-Ca.

## 2. Materials and Methods 

### 2.1. Sample Preparation

We examined two alloys with the respective compositions Mg–0.2 wt % Zn–0.5 wt % Ca and Mg–0.6 wt % Zn–0.5 wt % Ca. The focus was on the first alloy; the second was utilized to confirm the main findings. Commercially pure Mg (99.95%), Zn (99.5%), and Ca (99.5%) were used. The initial materials were cast, heat-treated, and finally hot extruded at 350 °C from 50 mm diameter to 6 mm for Mg-0.2Zn-0.5Ca, and 12 mm for Mg-0.6Zn-0.5Ca. Larger samples of Mg-0.2Zn-0.5Ca with a diameter of 20 mm were produced for tensile testing.

For HPT-processing, disc-shaped samples with a diameter of 6 mm (Mg-0.2Zn-0.5Ca) or 10 mm (Mg-0.6Zn-0.5Ca) and a thickness of 0.6 mm were cut from the extruded rods. In addition, discs of 7.5 mm diameter were produced for the tensile tests of HPT-processed Mg-0.2Zn-0.5Ca. The hydrostatic pressure applied during HPT was 4 GPa, and the torsion speed was 0.2 rot/min. The equivalent von Mises strain (*ε*) was calculated according to the following formula [18]:

(1) $$\varepsilon =\frac{1}{\sqrt{3}}\frac{2\pi Nr}{h}.$$

Here, *N* is the number of rotations, *r* the sample radius, and *h* the thickness of the sample. HPT-processing was mostly performed at room temperature (RT), but also at elevated processing temperatures between 96 °C and 235 °C. An inductive heating coil was used to heat both the anvils and the sample to the desired temperature, and an infrared light sensor controlled the temperature within ±1 °C. HPT was also performed at liquid nitrogen temperature (−196 °C). For this purpose, a container with liquid nitrogen was installed around the anvils and the sample.

Heat treatments of both the initial extruded and the HPT-processed samples were performed over different time periods and at different temperatures between 50 °C and 235 °C in an oil bath with a thermal stability of ±0.5 °C. After the heat treatments, the samples were dropped into the water at RT to ensure fast cooling and high accuracy of heat treatment duration. Heat treatments at higher temperatures of up to 465 °C were performed in a vacuum furnace. In the latter cases, quenching was not possible, and the cooling period was therefore longer.

### 2.2. Sample Characterization

Scanning electron microscopy was performed on selected samples using a Zeiss Supra 55 VP instrument equipped with backscattered electrons (BSE) and electron backscatter diffraction (EBSD) detectors. The samples were mechanically ground and polished, and a suspension of 0.3 µm alumina particles was used for the final polishing step. Transmission electron microscopy and scanning transmission electron microscopy (STEM) were performed with an FEI Talos F200X system of ScopeM, ETH Zurich on samples mechanically polished and ion milled (Gatan PIPS II of ScopeM, Zurich, Switzerland) at liquid nitrogen temperature to achieve electron penetrability. Chemical analysis in the STEM mode was conducted with the Talos Super-X EDS system. The Talos instrument was operated at 200 kV.

The microstructure of selected samples was investigated on the atomic level by atom probe tomography. Small rods with dimensions 0.3 × 0.3 × 6 mm³ were cut from the HPT-processed discs for further preparation via a two-step method [48]. After initial electropolishing of the samples with 10% perchloric acid and 90% methanol solution, 1% perchloric acid in butoxyethanol was applied as the second electrolyte. Measurements were performed on a LEAP^TM^ 4000 X HR atom probe (ScopeM) at a specimen temperature of −213 °C and with a pulse fraction of 20%, a pulse rate of 200 kHz, and a detection rate of 1% under ultra-high vacuum (<10^–10^ mbar) conditions. The software package IVAS 3.6.4^TM^ from Cameca (Gennevilliers, France) was deployed for the reconstruction procedure and analysis.

Differential scanning calorimetry (DSC) of HPT-processed samples was performed with a Netzsch DSC 204 using a heating rate of 10 °C/min. For Mg-0.2Zn-0.5Ca, a full HPT-disc was taken for the measurements, while for Mg-0.6Zn-0.5Ca a disc with a 6 mm diameter was cut from the HPT-disc off-centered via spark erosion. For each sample, the baseline was determined in a second heating run. The baseline was subtracted from the curve of the first heating to show the effects of microstructural changes on the heat flow.

The hardness of polished samples was measured using a Vickers indenter being part of a PAAR-MHT4 facility, by applying a load of 0.5 N for 10 s. The diagonals of the indents were measured with an optical microscope. To investigate the possible influence of texture, hardness was measured in the normal direction (ND) and in the transverse direction (TD) on related cross-sections of the discs. To achieve reliable average hardness values, at least 2 different samples were evaluated. Unless otherwise indicated, all hardness measurements were conducted at a sample radius of approximately 2 mm, and at least 10 indents per sample were evaluated. For Mg-0.6Zn-0.5Ca, an average value was calculated for the entire sample radius.

Strength and ductility were determined by tensile testing. Dogbone-shaped specimens were cut via spark erosion from initial extruded and HPT-processed discs. The cross-sectional area of the samples was on average 0.77 × 0.36 mm², and the parallel gauge length was 2.5 mm. The gauge length of the tensile samples was off-centered at a radius of the HPT-disc of approximately 2 mm. For the initial samples, the tensile axis was normal to the extrusion direction. The applied initial strain rate was 10^–3^ s^–1^. A micro-tensile test machine Messphysik ME30-1 equipped with a 1 kN load cell and a laser-speckle strain sensor was used. Thus, the strain could be measured directly, locally, and very accurately on the samples via the shift of objective laser-speckles. Details of this technique can be found elsewhere [49,50]. The stress-strain curves in the post-necking region were obtained from the displacement of the cross-head of the tensile test machine. To obtain average values for the tensile test results, 5 to 6 samples were tested for each condition. For the highly ductile extruded samples, all strain values beyond the yield point were obtained by the cross-head movement. This was necessary due to the development of a strong surface roughness during straining, which is detrimental for laser speckle strain measurements and resulted in a larger measurement uncertainty for these samples.

In addition to the HPT experiments, quenched samples of Mg-0.2Zn-0.5Ca were investigated. Discs of the extruded material with the same dimensions as those subjected to HPT were heated to 550 °C under an Ar atmosphere. After 60 min of annealing, they were quenched in water at RT. The hardness of the quenched samples was measured using a force of 0.2 N for 10 s. At least 4 different samples with approximately 30 indents per sample were evaluated for each condition investigated. In the case of the quenched samples, the indent direction was always the normal direction.

## 3. Results

### 3.1. Effect of HPT on Hardness and Microstructure of Mg-0.2Zn-0.5Ca

**Figure 1:**
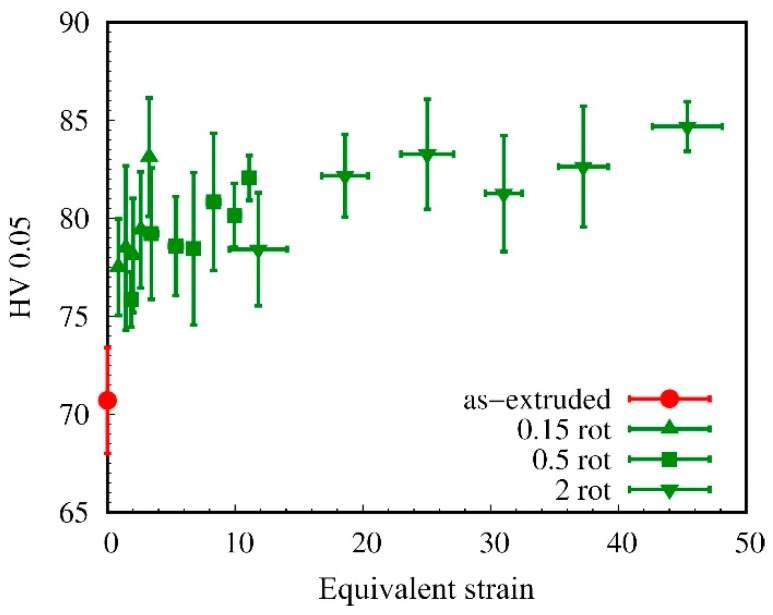
Hardness of high pressure torsion (HPT)-processed Mg-0.2Zn-0.5Ca as a function of equivalent von Mises strain. Measurements were performed in the normal direction (ND).

The hardness of HPT-processed Mg-0.2Zn-0.5Ca is shown in Figure 1 as a function of the equivalent strain during HPT-processing and compared to the as-extruded condition. Different numbers of rotations and measurement radii were investigated. It is obvious that—starting from the value of the initial extruded material (70.7 ± 2.7 HV)—the hardness increases with increasing HPT strain up to a saturation value of 83.0 ± 3.8 HV. This corresponds to an increase in hardness of 17%. These measurements were conducted in a normal direction (ND), i.e., in the extrusion direction and the direction of the HPT rotation axis, respectively. The hardness in the transverse direction (TD) was slightly different for the initial samples (68.8 ± 3.3 HV), indicating a texture that developed during the extrusion process. For HPT-processed samples, the average hardness in the saturation regime when measuring in TD was 83 ± 4 HV; thus, after HPT there was no difference anymore in the hardness in ND and TD. We can also conclude from space-resolved hardness measurements on cross-sections that the hardness was homogenously distributed throughout the sample thickness.

**Figure 2:**
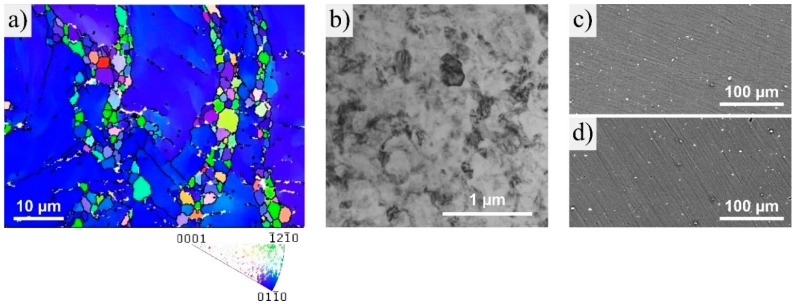
(**a**) Microstructure and corresponding inverse pole figure of extruded Mg-0.2Zn-0.5Ca as investigated by electron backscatter diffraction (EBSD); (**b**) STEM image of the microstructure after HPT-processing (2 rot); SEM-BSD images of (**c**) as-extruded and (**d**) HPT-processed (2 rot) Mg-0.2Zn-0.5Ca. All images were taken in ND.

EBSD investigations showed that the extruded alloy has a bimodal grain size distribution with larger grains in the range of 10–20 µm, plus small-sized grains with about 1–3 µm diameter; see Figure 2a. According to the STEM investigations shown in Figure 2b, HPT reduced the grain size down to the submicron range. Several contrasts also indicated that the microstructure exhibited a high concentration of deformation-induced lattice defects.

Another microstructural feature were the particles with a few micrometers in diameter, as shown in the SEM-BSD images in Figure 2c,d. Their size and distribution were not affected by HPT-processing. Further analysis in the STEM revealed that these particles consisted of calcium oxide, which is apparently harder than the Mg matrix and thus was not deformed during the HPT process.

### 3.2. Heat Treatment of HPT-Processed Mg-0.2Zn-0.5Ca

**Figure 3:**
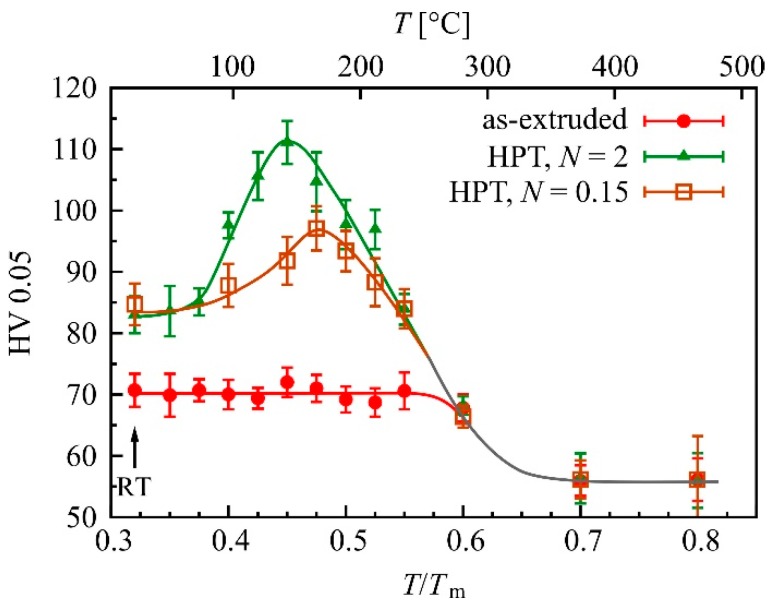
Hardness of initial extruded and HPT-processed Mg-0.2Zn-0.5Ca after heat treatments for 1 h. The lower caption gives the temperature *T* in terms of the melting temperature of Mg (*T*_m_), and the upper one in °C. Measurements were performed in ND; the solid lines are guides for the eye. ‘RT’ indicates room temperature.

Heat treatments lasting 1 hour were performed on the initial extruded and HPT-processed samples at 13 different temperatures between 0.35 and 0.8 *T*_m_ (melting temperature *T*_m_ of Mg: 650 °C). Two different degrees of deformation were investigated: A very low one (*N* = 0.15 rotations), and one in the saturation regime (*N* = 2 rotations). The development of hardness with temperature is shown in Figure 3. 

It can be seen that heat treatments are capable of significantly increasing the hardness of the HPT-processed Mg-0.2Zn-0.5Ca alloy. The hardness increased with increasing temperature up to a maximum and then decreased below the initial value until a final saturation occurred at temperatures beyond approximately 350 °C. At the larger HPT strain, the maximum hardness was higher and the corresponding temperature of the maximum was lower. In the extruded material no increase in hardness was observed.

**Figure 4:**
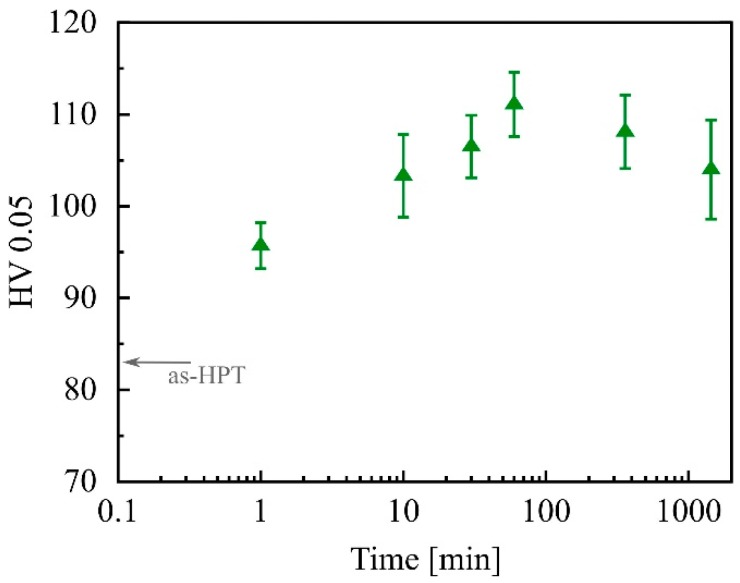
Hardness of HPT-processed (2 rot) and heat-treated Mg-0.2Zn-0.5Ca. The temperature was 0.45 *T*_m_ (142 °C), and the indent direction was equal to ND.

A further experiment was performed to investigate the influence of heat treatment duration. HPT-processed Mg-0.2Zn-0.5Ca was thermally treated at 0.45 *T*_m_ (142 °C) for six different time periods ranging from 1 min to 1440 min. The results are depicted in Figure 4. Even after 1 minute, the hardness increased from 83.0 ± 3.8 HV to 95.7 ± 2.5 HV. The maximum value (111.1 ± 3.5 HV) was reached after 1 h and was followed by a slight decrease over longer heat treatment periods.

The effect of a subsequent additional HPT deformation was also investigated. Samples with the maximum hardness were again HPT-processed and heat-treated (HT) a second time at 142 °C for 1 h. The hardness values are given in Table 1. We may conclude that the hardness increase due to heat treatment can be reversed by an additional HPT deformation but reappears with further heat treatment.

**Figure 5:**
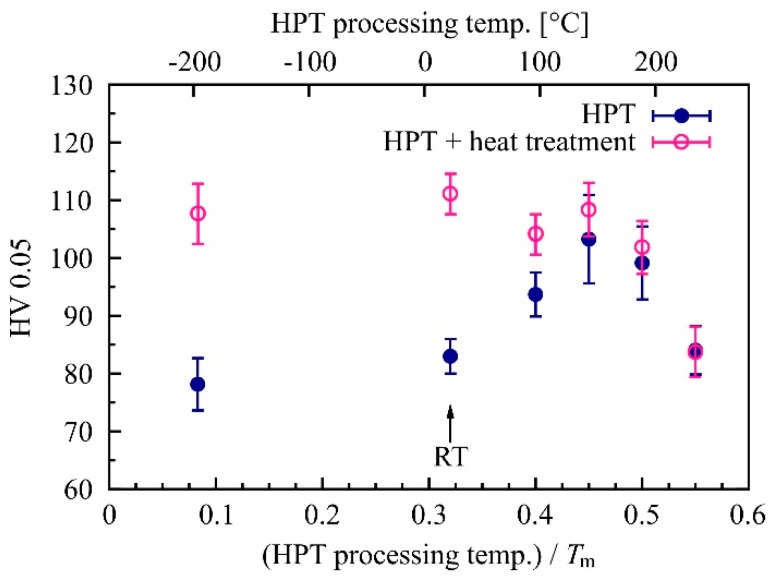
Hardness of HPT-processed Mg-0.2Zn-0.5Ca samples (2 rot, 4 GPa) as a function of HPT processing temperature. The second measurement series shows samples which were additionally heat-treated for 1 h at 142 °C after HPT.

More hardness measurements were performed to determine the effect of the processing temperature. Mg-0.2Zn-0.5Ca was HPT-processed at different elevated temperatures (96 °C, 142 °C, 189 °C, 235 °C), at room temperature, and at liquid nitrogen temperature (−196 °C). For each sample, two rotations were performed at a pressure of 4 GPa. Additional samples processed by HPT at different temperatures were subsequently heat-treated at 142 °C for 1 h. The results of these two series of measurements are shown in Figure 5. It can be seen that increased HPT temperatures have the same effect as post-HPT heat treatments. The peak hardness was reached at an HPT processing temperature of 142 °C with a value of 103.3 ± 7.7 HV. A further heat treatment (1 h/142 °C) of a sample processed at this temperature led to an additional slight increase in hardness up to a value of 108.4 ± 4.6 HV. HPT-processing at liquid nitrogen temperature, on the other hand, resulted in a lower hardness compared to room temperature processing. A post-HPT heat treatment, however, in this case, generated approximately the same high hardness value as did RT-HPT with the same subsequent heat treatment. Samples processed at temperatures beyond the hardness peak (i.e., above 0.45 *T*_m_) showed no or only very limited hardening capacity.

Several microstructural studies were performed to identify the origin of the hardness increase. TEM micrographs of the condition where the peak hardness was observed (2 HPT rotations + 1 h/142 °C) revealed an ultra-fine grained microstructure very similar to that of the as-HPT-processed state. After heat treatments above 0.6 *T*_m_ (duration 1 h), grain sizes in the micron-range were observed by optical microscopy. The grain sizes showed no significant differences, whether the material was in extruded or in HPT-processed condition before the heat treatments (0.6 *T*_m_: ~2–4 µm, 0.7 *T*_m_: ~10–12 µm, 0.8 *T*_m_: ~80–85 µm). This was also true for the corresponding hardness values (see Figure 3).

**Figure 6:**
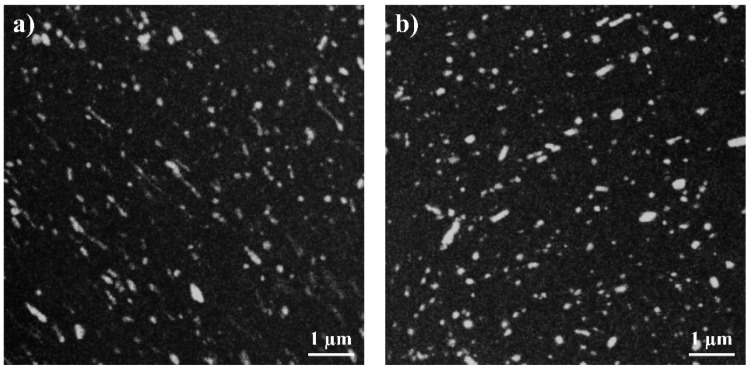
STEM/EDS images of Mg-0.2Zn-0.5Ca in (**a**) HPT-processed (2 rot, 4 GPa) and (**b**) HPT-processed and subsequently heat-treated condition. The bright areas indicate Ca-rich precipitates. Zn was homogeneously distributed and is therefore not shown in the images.

For a further microstructural investigation concerning the precipitation state of the samples, STEM/EDS was conducted on HPT-processed and on additionally heat-treated samples. Here, the heat treatment was performed at 150 °C for 10 min, resulting in a hardness of 104.2 ± 5.1 HV. Results of these investigations are shown in Figure 6. Ca-rich precipitates were detected in both conditions, while the distribution of Zn was homogeneous.

According to phase diagrams and the literature [51,52], we may conclude that (Mg,Zn)_2_Ca precipitates are present in the alloy. The average radius of the precipitates visible in Figure 6 was 38 nm for HPT-processed material, and 58 nm after the additional heat treatment (details of the evaluation performed by K. Werbach can be found elsewhere [53]). The average distances between the precipitates were 460 nm and 330 nm for specimens after HPT and HPT + HT, respectively. Thus, the heat treatment led to a slight increase in both the size and the density of the precipitates. Their appearance was also slightly more spherical after the heat treatment.

**Figure 7:**
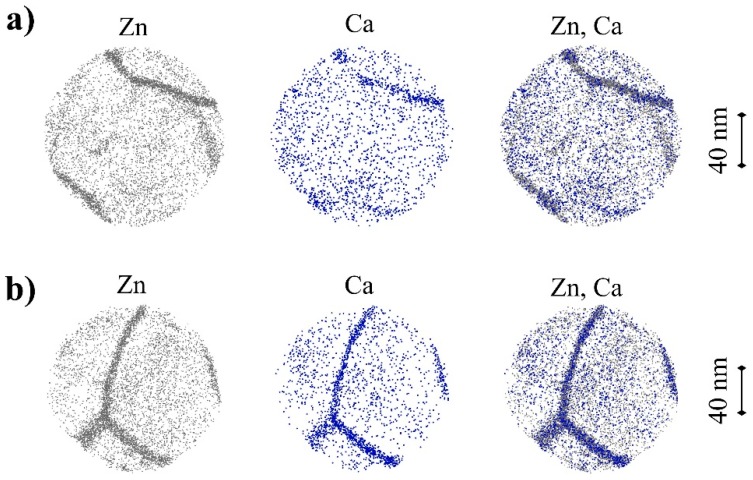
3D atom maps of Zn and Ca in Mg-0.2Zn-0.5Ca (**a**) after HPT-processing (2 rot, 4 GPa) and (**b**) after additional heat treatment (1 h/142 °C). Each disc (shown in top view) has a height of 15 nm; all Zn and Ca atoms detected are represented by grey and blue dots, respectively.

For microstructural analyses with even higher resolution, atom probe tomography (APT) was conducted on the two most important conditions. Exemplary results are depicted in Figure 7.

APT investigations reveal that on a very small scale the alloying elements Zn and Ca are not homogeneously distributed in the material. In fact, they are each segregated at the grain boundaries after both HPT-processing and the additional heat treatment. Among all the samples investigated, there is some tendency for grain-boundary segregation of Ca to be stronger after the heat treatment than after HPT only. However, a statistically significant quantification of this issue was not possible due to the differences between the atomic structures of the grain boundaries, their limited number in the small accessible sample volume, and the limited number of successful APT runs (five for HPT and seven for HPT and heat-treated condition). A further result of APT was that no nano-sized precipitates smaller than those shown in Figure 6 were detected in the grain interiors of any sample. An analysis of nearest-neighbor distances in three dimensions [54] of Zn and Ca revealed a slightly non-random distribution of the elements within the grain interior. However, this behavior was similar in both conditions, and APT indicated no additional clustering of Zn and Ca during the heat treatment.

### 3.3. HPT and Heat Treatment of Mg-0.6Zn-0.5Ca

As stated above, we aimed to confirm the main experimental findings obtained for HPT-processed Mg-0.2Zn-0.5Ca by investigating a second, similar alloy composition. TEM analyses revealed that this alloy, Mg-0.6Zn-0.5Ca, showed the same microstructural trends as the first one, e.g., an HPT-induced ultra-fine grained structure. The hardness of the initial extruded material was 75.1 ± 3.9 HV in ND and 66.4 ± 4.0 HV in TD, i.e., a bit higher than for Mg-0.2Zn-0.5Ca due to the higher alloy content. After HPT until saturation (two rotations) the hardness was increased by 17% up to 87.5 ± 5.6 HV for ND measurements. When comparing the hardness in TD, the increase due to HPT-processing was even larger (+36% up to 90.2 ± 3.5 HV). This again implies that after HPT-processing the difference between ND and TD measurements was no longer significant.

**Figure 8:**
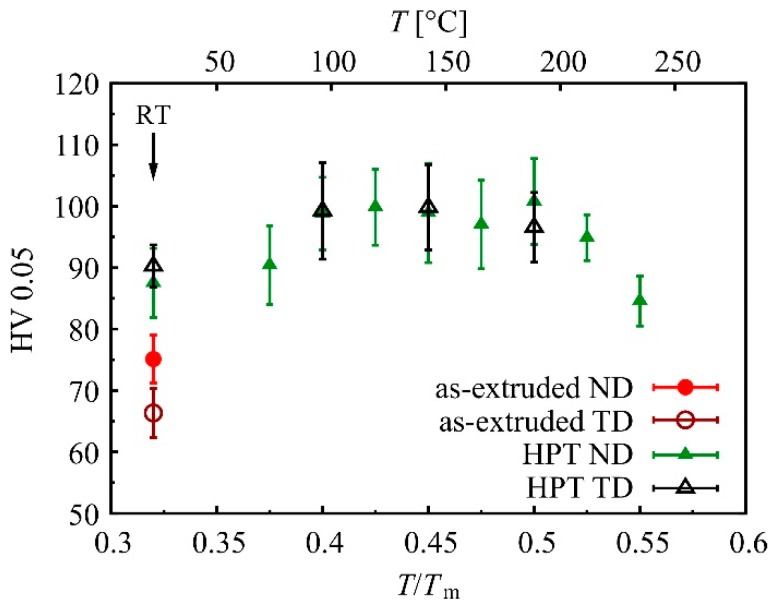
Hardness of Mg-0.6Zn-0.5Ca in the initial extruded state and after HPT (2 rot) and heat treatment for 1 h.

As with the first alloy, the hardness response upon 1 h of heat treatment at different temperatures was investigated. Results for measurement directions ND and TD can be seen in Figure 8. Very similar to the first alloy, post-HPT heat treatments lead to an increase in hardness. The hardness showed a broad maximum between 0.4 and 0.5 *T*_m_, and the increase was in the range of 9% to 13%. This means that the maximum was not as pronounced as in Mg-0.2Zn-0.5Ca.

### 3.4. DSC of HPT-Processed Mg-Zn-Ca Alloys

**Figure 9:**
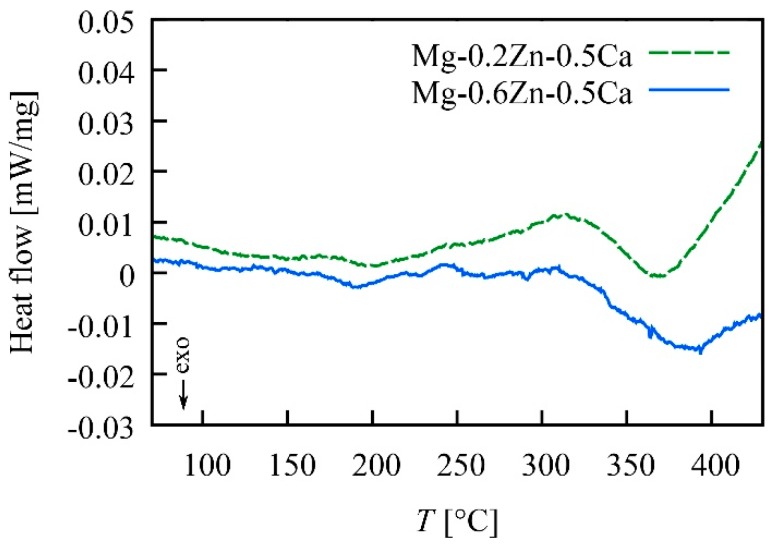
Differential scanning calorimetry (DSC) curves of the two investigated Mg alloys after HPT-processing (2 rot, 4 GPa).

DSC measurements were conducted on HPT-processed samples to investigate defect annealing behavior. In both alloys, an exothermic peak starting at ~320 °C was observed as can be seen in Figure 9. Along with the tremendous increase in grain size and the decrease in hardness observed in Mg-0.2Zn-0.5Ca after heat treatment in this temperature range (see Section 3.2), we may conclude that this peak was caused by the annealing of dislocations and grain boundaries.

### 3.5. Tensile Strength and Ductility of HPT-Processed and Heat-Treated Mg-Zn-Ca Alloys

**Figure 10:**
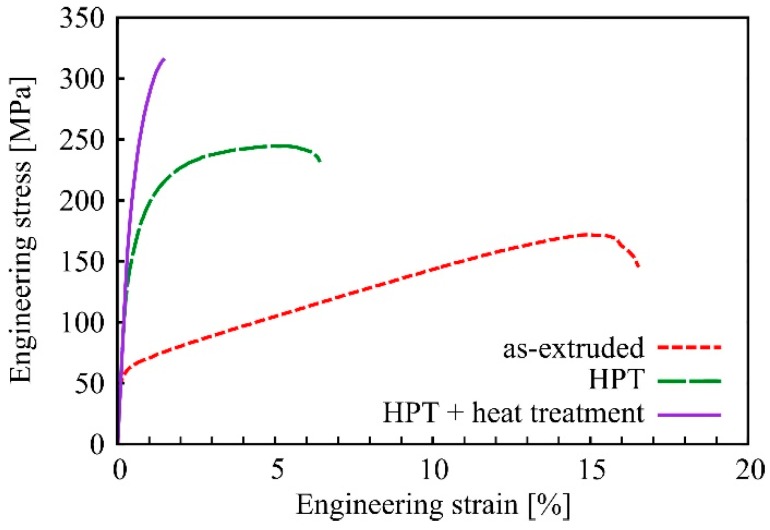
Exemplary tensile stress-strain curves of as-extruded, HPT-processed (2 rot), and HPT-processed and subsequently heat-treated (1 h/142 °C) Mg-0.2Zn-0.5Ca.

Tensile tests on Mg-0.2Zn-0.5Ca were conducted on the material which was extruded to a larger diameter (20 mm instead of 6 mm). The hardness of this as-extruded condition was, therefore, lower (62.8 ± 3.6 HV instead of 70.7 ± 2.7 HV when measuring in ND). However, after HPT-processing the same hardness was reached as for samples of 6 mm diameter. Exemplary engineering stress-strain curves obtained by micro-tensile testing are shown in Figure 10. It can be seen that the initial extruded material has a yield strength of approximately 65 MPa followed by a rather long period of strain hardening up to the ultimate tensile strength (UTS) of ~185 MPa at ~15% elongation. HPT-processing more than doubles the yield strength and also drastically increases the ultimate tensile strength by ~32%. However, strain hardening was less pronounced and the ductility was obviously lower than in the as-extruded material. The post-HPT heat treatment (condition of peak hardness) led to a further strong increase in both yield strength and ultimate tensile strength by 50% and 27%, respectively. However, the material was fairly brittle after the heat treatment. It shows only about 1% elongation to failure and nearly no strain hardening.

The average values for strength and ductility gained by the tensile tests are summarized in Table 2. The results for Mg-0.6Zn-0.5Ca are also specified there. This second alloy showed the same trends as the first. The increase in yield strength via HPT-processing was 66%. The difference compared to Mg-0.2Zn-0.5Ca was that, although upon heat treatment the hardness increased by 9%–13%, only an increase in yield strength but not in the ultimate tensile strength was observed. This is because the HPT-processed (and also the subsequently heat-treated) material is exceedingly brittle, and many samples fractured right after the elastic regime. In addition, this alloy showed no necking in the HPT-processed and in the heat-treated conditions, and brittle fracture occurs directly at the UTS.

### 3.6. Quenching and Heat Treatment of Mg-0.2Zn-0.5Ca

**Figure 11:**
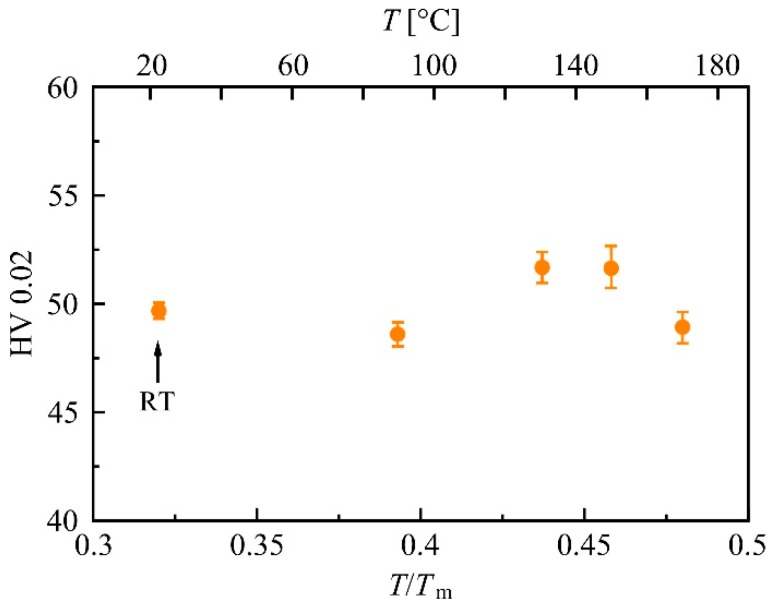
Hardness of annealed and quenched Mg-0.2Zn-0.5Ca after 10 min of heat treatment at different temperatures (90 °C, 130 °C, 150 °C, and 170 °C).

Samples of initial extruded Mg-0.2Zn-0.5Ca were annealed at 550 °C and quenched in water. The grain diameter was then evaluated from EBSD images (shown in [53]) as 140 ± 90 µm (average of 200 evaluated grains). The annealing resulted in a considerably lower hardness value when compared to the extruded material: 49.7 ± 0.3 HV instead of 70.7 ± 2.7 HV. After quenching, the samples were heat-treated, and the evolution of hardness with temperature is depicted in Figure 11. It can be seen that in the quenched alloy, an increase in hardness occurred at approximately the same temperature as in the HPT-processed material. Microstructural analyses shown in [53] revealed no indications of Mg_2_Ca precipitates in the as-quenched state or after the subsequent heat treatment.

## 4. Discussion

HPT-processing of low alloyed Mg-Zn-Ca alloys generates an increase in strength and hardness (see Figure 1, Figure 8 and Table 2) due to SPD-induced grain refinement (see Figure 2b). However, the most interesting observation was the additional substantial strength increase due to a post-HPT heat treatment, as shown in Figure 3 and Figure 8. For Mg-0.2Zn-0.5Ca the increase in hardness due to the heat treatment was even double the increase resulting from HPT-processing.

Our first assumption regarding the origin of the hardness increase was precipitation hardening [55]. Spatially resolved EDS scans in the STEM actually show some differences in the density and size of precipitates before and after heat treatment. The increase in yield strength (*Δσ*_Orowan_) due to precipitation hardening can be theoretically described by the following Orowan-type equation [56]:

(2) $$\Delta \sigma_{\mathrm{Orowan}}=M\;\frac{0.84\;G\;b}{2\pi \;{\left(1-\nu \right)}^{1/2}\;l}\;\ln\frac{D}{4b},$$

with *M* as the Taylor factor, *G* the shear modulus, *b* the Burgers vector, *ν* Poisson’s ratio, *l* the average distance between the precipitates, and *D* as the average precipitate diameter. Insertion of the material parameters for Mg (*M* = 4.2 for random texture [57], *G* = 17 GPa, *b* = 0.32 nm, *ν* = 0.35 [58]), and the values for *l* and *D* derived from Figure 6 (see Section 3.2) into Equation (2) leads to a difference in yield strength due to precipitation hardening of *Δσ*_Orowan, HPT+HT_ *− Δσ*_Orowan, HPT_ = 52 MPa − 34 MPa = 18 MPa between the two conditions investigated. The measured difference, however, was 79 MPa. Furthermore, the distance between the precipitates is of the same order of magnitude as the grain size and it can be expected that a significant amount of the precipitates is located at the grain boundaries. Therefore, the calculation using Orowan theory provides an upper limit for precipitation hardening. According to these considerations, the differences in the precipitation state cannot explain the extensive increase in strength. An investigation by Bamberger et al. [59] dealing with exactly the same material (Mg-0.2Zn-0.5Ca) also reports that the low alloy content does not permit precipitation hardening even after solution treatment. To further ascertain that precipitation hardening does not play an important role in our observations, we performed an analysis on the atomic scale using APT. From these results (Figure 7) we can exclude any formation of nano-sized precipitates during post-HPT heat treatment. Nevertheless, APT revealed that Zn and Ca accumulate at the grain boundaries in the HPT-processed and in the subsequently heat-treated condition. Grain boundary segregation has been observed in several SPD-processed materials [60] and may contribute to the specific properties of SPD materials [61,62]. However, in our case the change in grain boundary segregation due to thermal treatment was small, and it seems improbable that it causes an increase in hardness by 34%—a difference which is larger than that between extruded coarse-grained and HPT-processed ultra-fine grained condition. This conclusion is also supported by studies of Renk et al. [63,64], who investigated temperature-induced hardening in nanocrystalline austenitic steel and other materials. They found increased grain boundary segregation after heat treatment but could exclude this as the reason for the increased hardness: Different thermal treatments causing different amounts of segregation did not change the hardness.

However, we also cannot follow the explanation for the hardening phenomenon given in the above and other papers [65,66], which states that a dislocation starvation process—a reduction in the number of mobile dislocations due to heat treatment and a consequent enhancement of strength because further deformation requires dislocation emission from grain boundaries—is the reason for the increased hardness. The DSC analyses (Figure 9) of the two HPT-processed alloys showed an exothermic peak due to dislocation annealing and grain growth starting at ~320 °C. This temperature is far beyond the temperature where the increase in hardness is observed. TEM images also showed no indications of a reduced defect content. Thus, we conclude that in the heat-treated and hardened condition no significant dislocation annealing occurred.

Our explanation of the temperature-induced hardening of HPT-processed low alloyed Mg alloys is as follows: It is well known that SPD-processing generates an extraordinarily high concentration of vacancies [67,68,69,70]. During annealing, these vacancies become mobile and can form agglomerates [71]. In the case of Mg, disc-shaped agglomerates on the close-packed basal planes of the hexagonal lattice develop. If such a disc is large enough, it tends to collapse and produces a prismatic dislocation loop [72]. The Burgers vector of such a loop is perpendicular to the plane of the loop, and the dislocation loop is rather immobile. Even more important, the loops are located on the preferred slip plane of Mg and act as strong obstacles to the movement of other dislocations. Thus, the influence of dislocation loops on hardness and strength is markedly higher for Mg compared to other metals with different lattice structures, as for example fcc materials [72].

Pronounced loop hardening in Mg has been shown experimentally by Hampshire and Hardie [73]. They quenched 99.95% pure Mg from a temperature close to the melting point and thereby introduced a high vacancy concentration. A subsequent proper heat treatment (170 °C for 10 min) resulted in an increase in flow stress by more than 60%.

A theoretical description of loop hardening (increase in yield strength *∆σ*_loop_) of hexagonal metals was given by Kirchner [74]:

(3) $$\Delta \sigma_{\mathrm{loop}}=\;\frac{G\;b}{k}\;N^{a}d^{3a-1},$$

with *N* being the loop density (number of loops/m³), *d* the average loop diameter, and *a* and *k* constants, which depend on the ratio of loop distance (*λ = N*^−1/3^) to diameter. For a ratio of *λ*/*d* larger than approximately six, where the strain fields of the loops do not overlap, the constants amount to *a* = 1/2 and *k* = 0.122. For a smaller ratio, *a* = 4/3 and *k* = 0.001 [74]. The theory of Kirchner, on the one hand, provides a fit to the detailed experimental data of Hampshire and Hardie [73] but, on the other hand, analyzes the loop hardening in terms of the operating deformation mechanism. In the case of non-overlapping strain fields, the formula is in accordance with an Orowan-type of hardening, whereas in the case of smaller loop distances or larger loop diameters where the strain fields of the individual loops overlap, the theory suggests a direct overcoming of the loops by gliding dislocations to be the dominant mechanism [74].

Direct observation of the loops in HPT-processed and heat-treated Mg-Zn-Ca by means of TEM or STEM was not possible due to the small grain size and the generally high defect content in the HPT-processed alloy, both having contrasts not different enough from those of dislocation loops. However, in similar experiments (HPT-processing and subsequent heat treatment) on commercially pure Mg, we found a marked number of dislocation loops which were directly visible in STEM images [53]. Most of the observed loops had diameters between 10 and 100 nm, and a few even up to 200 nm.

According to Kirchner’s equation, loops with such diameters (10–100 nm) can account for the measured increase in yield strength of about 80 MPa when they are present with a density in the range of 10^–7^–10^–9^ nm^–3^. Assuming that a circular loop with diameter *d* consists of a total number of vacancies equal to $\frac{d^{2}\pi }{4\;b^{2}}$, this corresponds to a vacancy concentration between 5 × 10^–6^ and 1 × 10^–5^. Typical values of vacancy concentrations measured in HPT-processed materials are considerably larger (of the order of 10^–4^ [67,75,76]). During heat treatment, it is expected that a part of the vacancies anneals out while others form loops. Because of the high vacancy concentration after HPT-processing, the amount of vacancies forming loops can be smaller than 10% of the overall existing vacancies to explain the measured increase in strength.

To further verify the assumption that the loop hardening mechanism is the origin of the increase in strength observed in the HPT-processed and heat-treated Mg-0.2Zn-0.5Ca alloy, we performed quenching experiments on the same material. Quenching a material from solid solution at a temperature close to the melting point introduces a high density of thermal vacancies and vacancy agglomerates [77] but no additional dislocations or precipitates. Heat treatments of quenched, coarse-grained samples reveal an increase in hardness of at least 4% at the very same annealing temperature at which it occurs in HPT-processed samples (see Figure 11). This increase can only arise from quenched-in vacancies, which become mobile upon annealing. The effect is much smaller than after HPT-processing due to the fact that the typical vacancy concentration after HPT is exceptionally high and the samples for the quenching experiments had a thickness of at least 0.5 mm, which significantly limits the cooling rate during quenching and thereby the concentration of quenched-in vacancies.

Examples of hardening due to the agglomeration of deformation-induced vacancies have already been provided [71], recently also for SPD-deformed materials: Cengeri et al. [78] investigated HPT-processed Cu and Ni, and they observed thermally induced hardening in both materials, which does not arise from a change in dislocation density or crystallite size but from the agglomeration of deformation-induced vacancies. For ECAP Ni, Divinski et al. [79] made similar observations. They found an increase in hardness during heat treatments at 127 °C of ECAP-processed Ni. At the same temperature, an energy release during isothermal DSC was observed and, in combination with additional measurements of the temperature-dependent internal friction, the authors concluded that vacancy clusters form at this low temperature and cause the increase in hardness. The same phenomenon was also described by Su et al. [80], who observed an increase in yield stress in ECAP-processed commercially pure Al during heat treatment at 80 °C. At the same temperature, they found an exothermic peak in the DSC curve, which they ascribed to vacancies. Thus, they concluded that vacancies form clusters during heat treatment which pin the dislocations during subsequent deformation. In all the studies mentioned [78,79,80], the increase in hardness and yield strength was between 5% and 10% and thereby considerably lower than that observed in this study for Mg-Zn-Ca. This difference can—as already mentioned—be attributed to the hexagonal lattice of Mg, which makes loop hardening especially effective because of the coincidence of the loop planes with those of preferred dislocation slip.

Further investigations of the temperature-induced hardening phenomenon in HPT-processed Mg-0.2Zn-0.5Ca revealed that applying HPT-processing for a second time to already peak-hardened samples decreases the hardness to approximately the value before the heat treatment. However, it also restores the temperature hardening capacity of the material. Thus, it seems that a further HPT deformation destroys the dislocation loops formed during heat treatment but restores the high vacancy concentration, which is the prerequisite for a further hardness increase upon a second heat treatment. An additional experimental result was that increased HPT processing temperatures have the same effect as heat treatments after HPT (see Figure 5). This is also unexpected at first sight because an increase of deformation temperature usually decreases the strength due to larger grain sizes and decreased defect accumulation; see e.g., references [32,81]. Here, however, dislocation loops which increase the strength seem to form even during HPT-processing at elevated temperatures. Although some of the dislocation loops are destroyed during the continued deformation, others will remain in the material. Time also passes while the samples are cooled to RT after HPT, which allows additional loops to form.

It can be seen from the results of the tensile tests in Figure 10 and Table 2 that the temperature-induced hardening deteriorates the ductility of the materials. This effect of loop hardening on the ductility is well known from irradiation of metals, where both vacancies and dislocation loops are generated [82,83,84].

## 5. Conclusions

The investigations of two biodegradable low-alloyed Mg-Zn-Ca alloys showed that the hardness and strength of the materials were increased through HPT-induced grain refinement. A further significant and partially even greater increase was achieved by post-HPT heat treatment. This effect increases with increasing degree of HPT deformation and can also be reached within a one-step-process by increasing the HPT processing temperature.

Microstructural analyses combined with theoretical considerations, and parallel quenching experiments, led us to explain this behavior by the high density of vacancies after HPT. Because their diffusion is thermally activated, they form agglomerates and dislocation loops on the close-packed planes during heat treatment and thus act as strong obstacles for dislocation movement. This causes a remarkable increase in hardness and yield strength. By utilizing this strengthening mechanism in Mg-0.2Zn-0.5Ca, a particularly high ultimate tensile strength of more than 300 MPa can be reached. Low-alloyed Mg alloys with interesting biodegradation properties can thus be further optimized by this new hardening process, favoring their use as material for biodegradable load-bearing medical implants.

## Figures and Tables

**Table 1:** Hardness after different combinations of HPT deformation and heat treatment.

Condition	Hardness [HV 0.05]
HPT (2 rot)	83.0 ± 3.8 HV
HPT (2 rot) + HT (1 h/142 °C)	111.1 ± 3.5 HV
HPT (2 rot) + HT (1 h/142 °C) + HPT (2 rot)	86.6 ± 4.6 HV
HPT (2 rot) + HT (1 h/142 °C) + HPT (2 rot) + HT (1 h/142 °C)	104.6 ± 3.7 HV

**Table 2:** Average values and standard deviations of yield strength (*σ*_0.2_), ultimate tensile strength (UTS), uniform elongation (*ϵ*_unif._) and total elongation (*ϵ*_total_) of Mg-0.2Zn-0.5Ca and Mg-0.6Zn-0.5Ca in different conditions.

Mg-0.2Zn-0.5Ca	**Condition**	***σ*_0.2_ [MPa]**	**UTS [MPa]**	***ϵ*_unif._ [%]**	***ϵ*_total_ [%]**
	as-extruded	64 ± 5	184 ± 11	15 ± 5	16 ± 5
	HPT (2 rot)	158 ± 9	242 ± 3	4.3 ± 1.0	5.3 ± 1.6
	HPT (2 rot) + 1 h/142 °C	237 ± 10	308 ± 20	1.0 ± 0.6	1.1 ± 0.7
Mg-0.6Zn-0.5Ca	**Condition**	***σ*_0.2_ [MPa]**	**UTS [MPa]**	***ϵ*_unif._ [%]**	***ϵ*_total_ [%]**
	as-extruded	117 ± 5	237 ± 3	14 ± 2	15 ± 3
	HPT (2 rot)	194 ± 8	247 ± 11	1.4 ± 0.6	1.4 ± 0.6
	HPT (2 rot) + 1 h/190 °C	216 ± 16	238 ± 33	0.4 ± 0.4	0.4 ± 0.4
